# A Serine Protease Isolated from the Bristles of the Amazonic Caterpillar, *Premolis semirufa*, Is a Potent Complement System Activator

**DOI:** 10.1371/journal.pone.0118615

**Published:** 2015-03-11

**Authors:** Isadora Maria Villas Boas, Giselle Pidde-Queiroz, Fabio Carlos Magnoli, Rute M. Gonçalves-de-Andrade, Carmen W. van den Berg, Denise V. Tambourgi

**Affiliations:** 1 Immunochemistry Laboratory, Butantan Institute, São Paulo, SP, Brazil; 2 Institute of Molecular and Experimental Medicine, School of Medicine, Cardiff University, Cardiff, United Kingdom; University of Leicester, UNITED KINGDOM

## Abstract

**Background:**

The caterpillar of the moth *Premolis semirufa*, commonly named pararama, is found in the Brazilian Amazon region. Accidental contact with the caterpillar bristles causes an intense itching sensation, followed by symptoms of an acute inflammation, which last for three to seven days after the first incident. After multiple accidents a chronic inflammatory reaction, called “Pararamose”, characterized by articular synovial membrane thickening with joint deformities common to chronic synovitis, frequently occurs. Although complement mediated inflammation may aid the host defense, inappropriate or excessive activation of the complement system and generation of anaphylatoxins can lead to inflammatory disorder and pathologies. The aim of the present study was to evaluate, *in vitro*, whether the *Premolis semirufa*’s bristles extract could interfere with the human complement system.

**Results:**

The bristles extract was able to inhibit the haemolytic activity of the alternative pathway, as well as the activation of the lectin pathway, but had no effect on the classical pathway, and this inhibition seemed to be caused by activation and consumption of complement components. The extract induced the production of significant amounts of all three anaphylatoxins, C3a, C4a and C5a, promoted direct cleavage of C3, C4 and C5 and induced a significant generation of terminal complement complexes in normal human serum. By using molecular exclusion chromatography, a serine protease of 82 kDa, which activates complement, was isolated from *P. semirufa* bristles extract. The protease, named here as Ps82, reduced the haemolytic activity of the alternative and classical pathways and inhibited the lectin pathway. In addition, Ps82 induced the cleavage of C3, C4 and C5 and the generation of C3a and C4a in normal human serum and it was capable to cleave human purified C5 and generate C5a. The use of Phenanthroline, metalloprotease inhibitor, in the reactions did not significantly interfere with the activity of the Ps82, whereas the presence of PMSF, serine protease inhibitor, totally blocked the activity.

**Conclusion:**

These data show that a serine protease present in the *Premolis semirufa*’s bristles extract has the ability to activate the complement system, which may contribute to the inflammatory process presented in humans after envenomation.

## Introduction

The caterpillar of the moth *Premolis semirufa* (Walker, 1856), commonly named pararama, belongs to the Arctiidae family and it is found in the Brazilian Amazon region feeding on the rubber tree *Hevea brasiliensis*. Accidental contact with the caterpillar bristles generally causes an intense itching sensation, followed by symptoms of an acute inflammation such as pain, heat and redness, which last for three to seven days after the first incident [1,2]. However, a chronic inflammatory reaction, characterized by articular synovial membrane thickening with joint deformities common to chronic synovitis, frequently occurs in individuals after multiple accidents [3]. This chronic inflammatory reaction is called “Pararamose” and due to its importance as an occupational disease, predominantly in the rubber tree areas of Pará, Brazil, this caterpillar envenomation—“Pararama associated phalangeal Periarthritis”—was included into the “Manual of diagnosis and treatment of envenomations”, by the Brazilian Ministry of Health in 1992 [4]. So far, there is no effective treatment for accidents with pararama.

Studies about “pararamose” are scarce not only regarding the characterization of the toxic substances released by the caterpillar bristles, but also on the molecular mechanisms involved in its pathogenesis. Recently, we have demonstrated that *Premolis semirufa* caterpillar bristles’ crude extract presents strong proteolytic activity and it can induce an intense inflammatory process, characterized by the presence of neutrophils in the paw tissues of injected mice and a strong, specific antibody response [5]. Moreover, in a subsequent study we confirmed that the strong cellular and humoral immune responses induced by bristles toxic components in a murine model, consisted of a intense infiltration of neutrophils and macrophages to the envenomation site, proliferation/migration and activation of T and B lymphocytes to the draining the lymph nodes and elevated plasma levels of IL-6, IL-10, IL-12, IL-17 and IL-23 [6].

The complement system, a first-line of innate immune defense against invading pathogens, has presented several additional roles such as, modulation of adaptive immune response, elimination of immune complexes and apoptotic cells, angiogenesis, tissue regeneration, and organogenesis [7–9]. It comprises about 30 fluid phase molecules and several membrane inhibitors or receptors for complement components. Complement activation on surfaces may be initiated through one or more of three pathways, the classical, lectin, or alternative pathways, all of which converge at the level of C3 activation. C3 is the most abundant complement protein and its cleavage and the stable, covalent linkage of its fragments to target surfaces is a critical outcome of complement activation [10].

Complement amplification leads to the formation of C5 convertases, which cleave C5 into the anaphylatoxin C5a and fragment C5b, which, in turn, when associated to C6, C7 and C8, inducing the binding of several units of C9, form a lytic pore, the membrane attack complex (MAC) [11].

Anaphylatoxins (C3a, C4a and C5a) are potent inflammatory mediators targeting a broad spectrum of immune and non-immune cells and have a number of crucial roles in immune responses and inflammation, such as to regulate vasodilation, to increase the permeability of small blood vessels, and to induce contraction of smooth muscles. C3a, and especially C5a, are powerful chemoattractants that guide neutrophils, monocytes and macrophages toward sites of complement activation [12].

Although complement mediated inflammation may aid the host defense, inappropriate or excessive activation of the complement system and generation of anaphylatoxins can lead to inflammatory disorder and pathologies, with tissue damage in certain pathological conditions [13, 14]. Based on this information, and considering that the *P*. *semirufa* bristles extract has proinflammatory activity, as seen in a murine model, the aim of the present study was to evaluate, *in vitro*, whether the *Premolis semirufa*’s bristles extract could interfere with the human complement system.

## Materials and Methods

### Chemicals, reagents and buffers

Bovine serum albumin (BSA), ortho-phenylenediamine (OPD), 1,10-phenanthroline (Phen), ethylene glycol-bis-(beta-aminoethylether)-N,N,N',N'- tetraacetic acid (EGTA), Triton X-100, gelatin, Coomassie Brilliant Blue R-250, mannan and human IgM antibodies were purchased from Sigma (St. Louis, MI, USA). Tween 20 and Phenylmethylsulfonyl fluoride (PMSF) were purchased from Labsynth (Diadema, SP, Brazil) and Boehringer Ingelheim (Ridgefield, CT, USA). Rabbit anti-goat (RAG) IgG labelled with horseradish peroxidase (IgG-HRPO) were from Pierce (Rockford, IL, USA). Purified human C3, C4, C5 and goat IgG anti-human C4 were from Quidel Corporation (San Diego, CA, USA). The following buffers were used: Alsever’s Solution, pH 6.1, 114 mM citrate, 27 mM glucose, 72 mM NaCl; Veronal-Buffered Saline (VBS^2+^), pH 7.2, 2.8 mM barbituric acid, 145.5 mM NaCl, 0.8 mM MgCl_2_, 0.3 mM CaCl_2_, 0.9 mM sodium barbital; BVB^2+^ (VBS^2+^, pH 7.2, containing 0.5 mM MgCl_2_, 0.2 mM CaCl_2_, 0.1% BSA and 0.05% Tween 20); Alternative Pathway (AP) buffer, pH 7.4, 5 mM sodium barbital, 10 mM EGTA, 7 mM MgCl_2_ and 150 mM NaCl; Phosphate-Buffered Saline (PBS), pH 7.2, 8.1 mM sodium phosphate, 1.5 mM potassium phosphate, 137 mM sodium chloride and 2.7 mM potassium chloride; Zymography buffer, pH 8.3, 50 mM Tris-HCl, 200 mM sodium chloride, 10 mM calcium chloride and 0.05% Brij-35 P.

### Extract of caterpillar bristles

Caterpillars from *Premolis semirufa* were collected in non-protected areas of the city of São Francisco do Pará, Pará, Brazil {{Coord|1|10|08.7|S|47|47|26.3|W|}}. The license for capture, transportation and maintenance of the animals were provided by Chico Mendes Institute for Biodiversity Conservation (ICMBIO) of the Brazilian Ministry of the Environment—permission no. 11971–2 and maintained at the Immunochemistry Laboratory, Butantan Institute, SP, Brazil. The bristles extract was prepared after incubating the caterpillars at 4°C for a few minutes; the bristles were cut off with scissors at the point of insertion in the tegument, avoiding any tegument incision, and then suspended in cold phosphate-buffered saline (PBS). This suspension was macerated with a glass stick, homogenised and centrifuged at 560 × *g* for 20 min at 4°C. The supernatant was collected, and its protein content was determined using the BCA Protein Assay Kit (Pierce Biotechnology, Rockford, IL, USA). Supernatant aliquots were stored at-80°C until use. Authorisation to access the venom of the *Premolis semirufa* caterpillar was provided by the Brazilian Institute of Environment and Renewable Natural Resources (IBAMA), an enforcement agency of the Brazilian Ministry of the Environment (permission no. 01/2009).

The concentration of lipopolysaccharides (LPS) in the samples of *Premolis semirufa*’s bristles extract was evaluated by the Limulus Amebocyte Lysate (LAL) test in the Section of Microbiological Control of the Butantan Institute (Service of Quality Control—Bioindustrial Division), with the PYROGENT Plus Gel Clot LAL Assays kit (Lonza, Walkersville, MD, USA), according to the manufacturer's specifications. The concentration of endotoxin, calculated using a standard curve of LPS from *E*. *coli* (2.5 to 0.125 EU/mL), showed values below the limit of *detection*, i.*e*., 0.125 EU/mL; thus, all of the effects observed in our experiments resulted from the components present in the extract.

### Fractionation of *Premolis semirufa*’s bristles extract

*Premolis semirufa*’s bristles extract samples (1 mg/ sample) was applied to a FPLC-GP-250 Plus system using a molecular exclusion column Superdex 200 10/300 GL (GE Healthcare, PA, USA), equilibrated with 0.05 M ammonium bicarbonate, pH 7.4. Elution was carried out using the same buffer at a flow rate of 30 mL/h and the chromatographic profiles monitored by ultraviolet absorption (280 nm). Fractions (5 mL) from the protein peaks were collected, lyophilized and resuspended in pyrogen-free saline (1.0 mL).

### Normal human serum, erythrocytes and ethics statement

Blood samples drawn to obtain sera were collected without anticoagulant and allowed to clot for 4 h at 4°C. After centrifugation at 560 × *g* for 15 min at 4°C, the normal human serum (NHS) was collected and immediately frozen at-80°C until use. The human blood samples in this study were obtained from healthy donors who clearly knew the purpose of the project and signed the corresponding informed consent form. This study was approved by the Human Research Ethics Committee (HREC) from ABC Foundation School of Medicine, Santo André, Brazil (CAAE 02193312.0.3001.5467) and from Institute of Biomedical Sciences at the University of São Paulo, São Paulo, Brazil (no. 155.621).

Blood samples from sheep and rabbit, drawn to obtain erythrocytes (E) for subsequent use as target cells in complement assays, were collected in anticoagulant Alsever’s solution. The protocol was in accordance with the ethical principles in animal research adopted by the Brazilian Society of Animal Science and the National Brazilian Legislation no.11.794/08. The protocol was approved by the Animal Care and Use Committee from Butantan Institute (permission no. 413/07).

### Effect of *Premolis semirufa*’s bristles extract/fraction on complement activity

Samples of the normal human serum (NHS), as source of complement components, were incubated with various concentrations of the *Premolis semirufa*’s bristles extract/fraction, PBS or BVB^2+^, as control, for 30 min or 1 h at 37°C, and then assayed for remaining C-activity of any of the three pathways or measurement of anaphylatoxins.

**a. Haemolytic measurement of the complement activity.** For classical pathway analysis, sheep erythrocytes (E^S^) were washed in PBS and a 2% suspension was incubated with an equal volume of a 1:500 dilution in PBS of rabbit anti-sheep erythrocyte serum for 30 min at 37°C. The antibody-sensitized sheep erythrocytes were washed and resuspended in VBS^2+^ at 2% final concentration. For alternative pathway analysis, rabbit erythrocytes (E^R^) were washed in PBS and resuspended in AP buffer at 2% final concentration. The antibody-sensitized sheep erythrocytes or the rabbit erythrocytes (50 μl) were added to each well, of a 96-well plate, and incubated, for 30 min at 37°C, with the extract-, fractions or PBS- treated NHS samples, VBS^2+^ or AP buffer (background lysis) or with deionised water (100% lysis) at final volume 150 μl/well.

Unlysed cells were removed by centrifugation at 304.2 × *g* at 4°C for 5 min. Fifty microliters of each supernatant were transferred to new 96-well plates containing 200 μL of water, and the absorbance was measured in an ELISA reader (Multiskan spectrophotometer EX, Labsystems, Finland) at λ 414 nm as an index of haemolysis. Percentage of haemolysis for each well and the number of CH_50_ and AP_50_ for each serum were calculated by standard methods [15].

**b. Determination of the classical and lectin pathways activation by ELISA.** ELISA plates (Costar, Corning Inc., USA) were coated with 2 μg/mL of human IgM (for classical pathway) or 10 μg/well of Mannan (for lectin pathway), overnight at 4°C, washed three times with PBS/0.05% Tween 20, and blocked using 1% BSA in PBS for 3 h at 37°C. After washing, serial dilutions of NHS samples treated with BVB^2+^ or *Premolis semirufa*’s bristles extract/fraction, in the presence or absence of 1,10-Phenanthroline were added. After 1 h of incubation at 37°C, plates were washed with BVB^2+^ and incubated with anti-human C4 (1:1,000) for 1 h at 37°C. Plates were washed three times with BVB^2+^/Tween 0.05% and incubated with the specific anti-IgG antibody conjugated with HRPO (1:5,000) for 1 h at 37°C. Plates were washed and the reactions developed with OPD substrate according to conditions established by the manufacturers (Sigma). The absorbances were recorded in an ELISA reader (Multiskan spectrophotometer EX, Labsystems, Finland) at λ 492 nm. For complement activity ELISA, normal human serum, arbitrarily set at 1000 arbitrary units (aU)/mL, was used to produce a calibration curve [15].

**c. Analysis of anaphylatoxins production in NHS incubated with the Premolis semirufa’s bristles extract/fraction.** Samples of NHS (50 μL) were incubated with the *Premolis semirufa*’s bristles extract (175 μg/mL), fraction (0.33 μg/mL) or PBS, in the presence or absence of 1,10-Phenanthroline (10 mM), for 30 min at 37°C, and C3a/C3a-desArg, C4a/C4a-desArg and C5a/C5a-desArg generation was measured using the Human Anaphylatoxin Cytometric Bead Array—CBA (BD Biosciences PharMingen, San Jose, CA, USA), according to the manufacturer's instructions. Two-color flow cytometric analysis was performed using a FACSCalibur flow cytometer (Becton & Dickinson Immunocytometry Systems, CA, USA). Data were acquired and analyzed using Becton Dickinson Cytometric Bead Array CBA Analysis Software. Anaphylatoxin concentrations (ng/mL) were determined from the standard curves, plotting Anaphylatoxin concentration *versus* Median Fluorescence Intensity. Alternatively, the anaphylatoxins generation was determined by using Human C3a, C4a and C5a ELISA Kits (BD OptEIA—BD Biosciences, San Jose, CA, USA), according to the manufacturer's instructions.

Samples of NHS (50 μL) were incubated with the *Premolis semirufa*’s bristles extract (175 μg/mL), fractions (0.5 μg/mL) or PBS, in the presence or absence of 1,10-Phenanthroline (10 mM), for 30 min at 37°C and SC5b-9 complex (TCC), present in human serum samples, was measured using the MicroVue SC5b-9 Plus EIA Kit (Quidel Corporation, San Diego, CA, USA), according to the manufacturer's instructions. SC5b-9 complex concentrations (ng/mL) were determined from the standard curves, plotting SC5b-9 complex concentration *versus* Absorbance at 450 nm.

### Proteolytic activity of the *Premolis semirufa*’s bristles extract/fractions on the Complement system components

Samples of the extract (2.0 μg for C3 and C4 or 3.0 μg for C5) or of Ps82 (0.1 μg) were incubated with 3.0 μg of the purified human components C3, C4 and C5 (Quidel Corporation, San Diego, CA, USA) at 37°C for 1 h, in the presence or absence of 10 mM 1,10-Phenanthroline or PMSF, metallo- and serine-protease inhibitors, respectively. The cleavage of the C-components was visualized in 10% SDS-PAGE gels, developed under reducing conditions [16]. Molecular weight markers were included in all runs and gels were stained with silver [17].

Alternatively, samples of human C5 were incubated with the Ps82 for 30 min at 37°C, in the presence or absence of PMSF, and evaluated for the generation of C5a/C5a desArg using the Human C5a ELISA Kit (BD OptEIA—BD Biosciences, San Jose, CA, USA), according to the manufacturer's instructions.

### Proteolytic activity analysis by gelatin Zymography

Samples of *Premolis semirufa*’s bristles extract (0.5 μg) or fraction (0.05 μg) were incubated, at 37°C for 30 min, in the presence or absence of 10 mM 1,10-phenanthroline or PMSF, metallo- and serine-protease inhibitors, respectively, solubilised in non-reducing sample buffer and separated on 10% SDS-PAGE gels containing 1 mg/mL gelatin. The gels were washed, for 30 min at room temperature in 2.5% Triton X-100, and incubated for 12 h at 37°C in Zymography buffer. Following incubation, the gels were stained with 0.2% Coomassie Brilliant Blue R-250 and the gelatinolytic activity was detected as unstained bands, according to the established method described by Kleiner and Stetler-Stevenson [18], with some modification.

### Statistical analyses

One-way ANOVA with Bonferroni post-tests were used to evaluate significant differences between control and experimental results. Statistical analysis was performed using GraphPad Prism software. Differences were considered statistically significant when *p* values were *p*<0.05, *p*<0.01 and *p*<0.001.

## Results

### Action of the *Premolis semirufa*’s bristles extract on the complement system activation pathways

**Fig 1:**
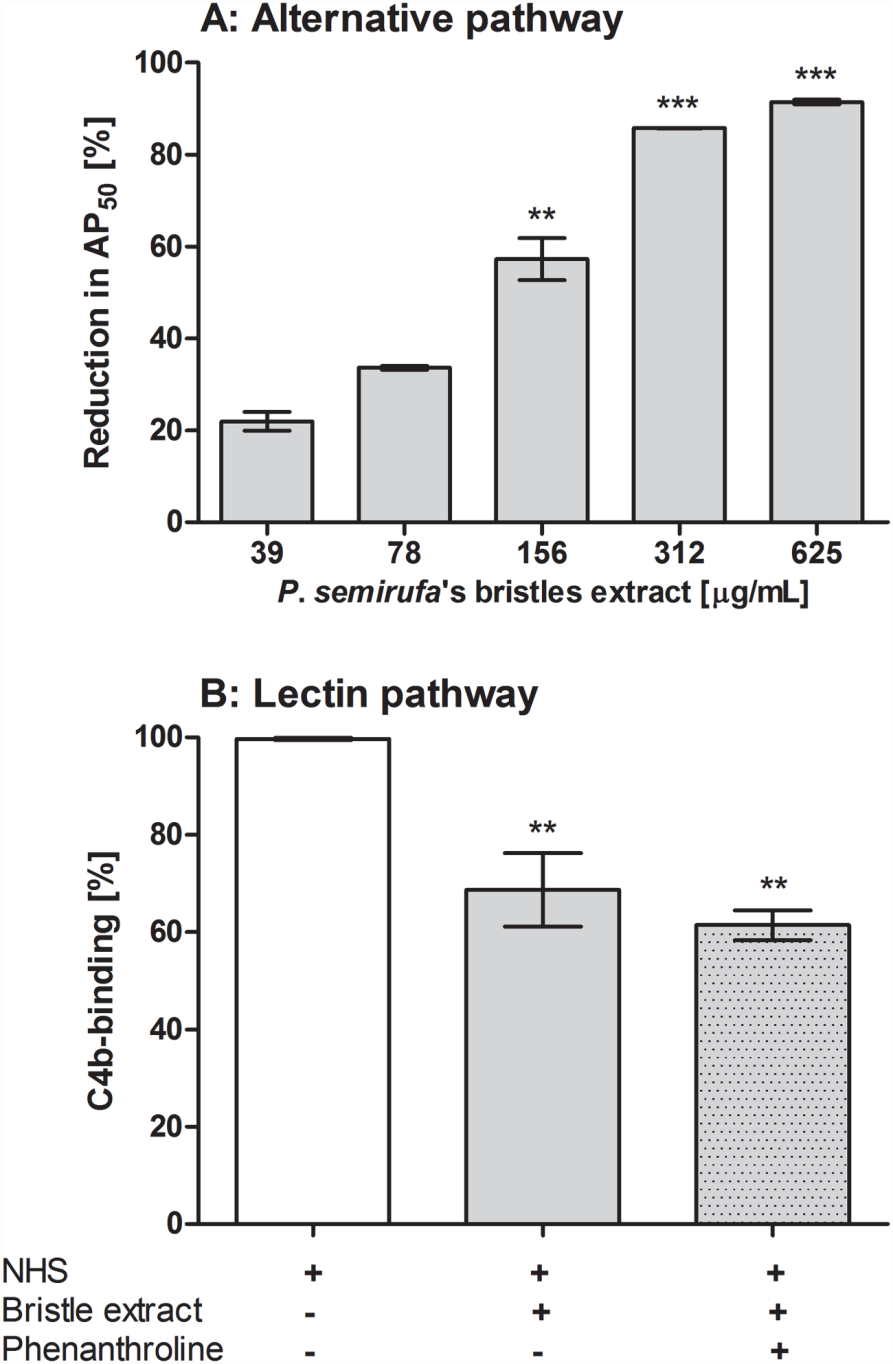
Activity of the *Premolis semirufa’s* bristles extract on the alternative and lectin pathways. **[A]** Samples (50 μL) of normal human serum (NHS) were pre-incubated with increasing concentrations of the bristles extract or PBS, at 37°C for 30 min, and the residual haemolytic activity of the mixtures was measured. **[B]** The residual lectin pathway complement activity was assessed by incubation of NHS with the bristles extract (175 μg/mL), in the presence or absence of 1,10-Phenanthroline (10 mM), at 37°C for 30 min, and added to plates coated with mannan (100 μg/mL). Data are representative for three separate experiments, performed in duplicate, and the results are expressed as percentage of AP_50_ reduction (%) ± SD (for A) and percentage of C4b deposition (%) ± SD (for B). (*) *p*<0.05, (**) *p* < 0.01 and (***) *p* < 0.001: significant differences between the mean values obtained with PBS and the treatments.

In order to evaluate the possible action of *Premolis semirufa*’s bristles extract on the complement system, samples of normal human serum were incubated with PBS or the extract and the remaining complement lytic activity was measured under conditions to develop the alternative, lectin or classical pathways. Fig. 1A shows that the extract induced a significant, dose-dependent, reduction in the haemolytic activity of the alternative pathway. Furthermore, analysis of the activity of the extract (at 175 μg/mL) on the lectin pathway also showed a significant reduction, as measured by the deposition of C4b on mannose-coated plates. The metalloprotease inhibitor 1,10-phenanthroline did not inhibit the action of the extract on lectin pathway (Fig. 1B). No action of the extract on the classical pathway was observed (data not shown).

### Induction of the generation of the anaphylatoxins in human serum by the *Premolis semirufa*’s bristles extract

**Fig 2:**
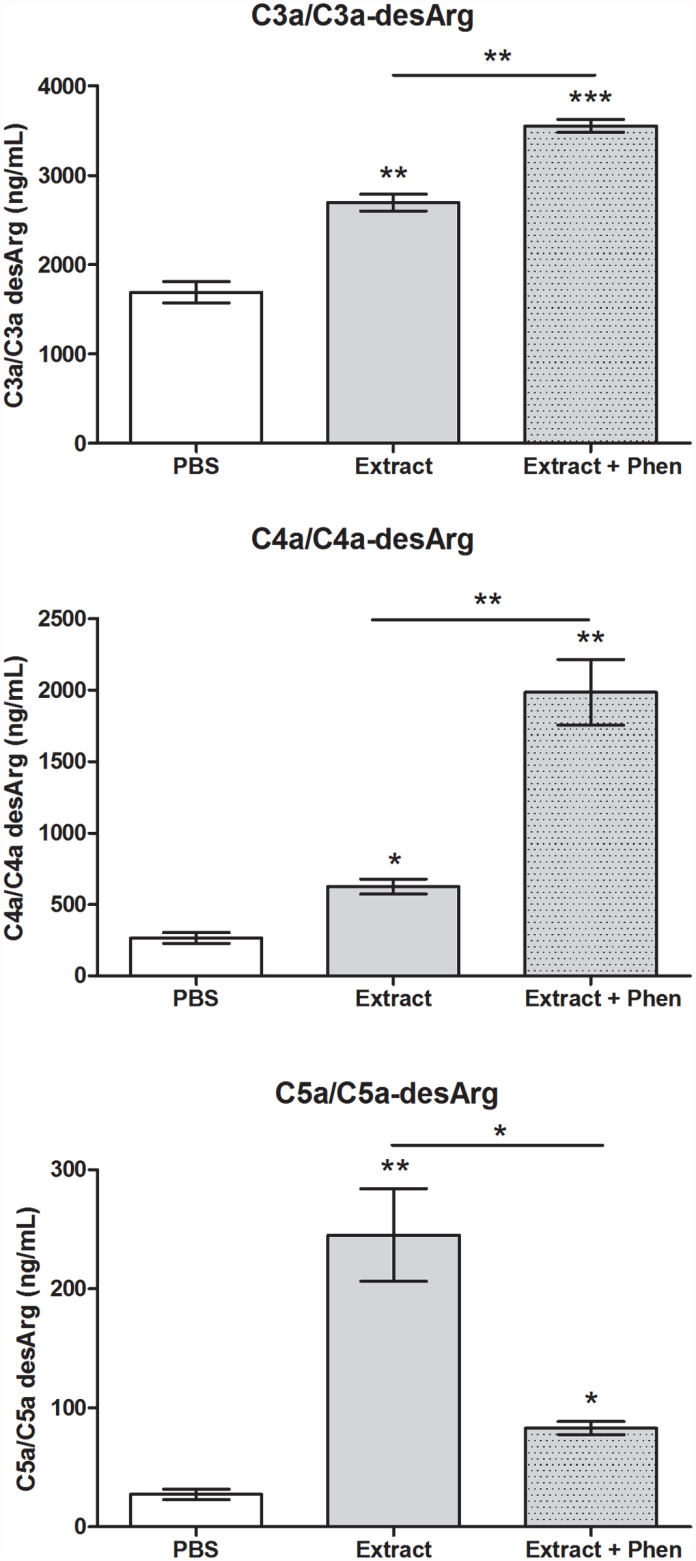
Human serum anaphylatoxins generation induced by the *Premolis semirufa’s* bristles extract. Samples of NHS (50 μL) were incubated with the bristles extract (175 μg/mL) or PBS, in the presence or absence of 10 mM 1,10-Phenanthroline (Phen), at 37°C for 30 min. The generation of the anaphylatoxins (C3a, C4a and C5a) was measured using the *Human Anaphylatoxin Cytometric Bead Array* (CBA). Data are representative for two separate experiments, performed in duplicate, and the results are expressed as concentration of each anaphylatoxin per mL of human serum (ng/mL) ± SD. (*) *p* < 0.05 and (**) *p* < 0.01: significant differences between the mean values obtained with the buffer and between the treatments.

To determine whether the reduction in complement activities observed above was caused by inhibition or activation/consumption of complement, the ability of the bristles extract to induce the generation of complement activation products, the anaphylatoxins, in normal human serum was assessed. Fig. 2 shows that the *Premolis semirufa*’s bristles extract induced the production of significant amounts of all three anaphylatoxins. The addition of 1,10-phenanthroline, a metalloprotease inhibitor, inhibited the generation of C5a/C5a-desArg, while it increased the generation of C3a/C3a-desArg and C4a/C4a-desArg. These data suggest that the reduction in the alternative and lectin pathways, as observed above, is due to activation/consumption of the complement rather than inhibition and that metalloproteases may be involved.

### Induction of the generation of Terminal Complement Complex in human serum by the *Premolis semirufa*’s bristles extract

**Fig 3:**
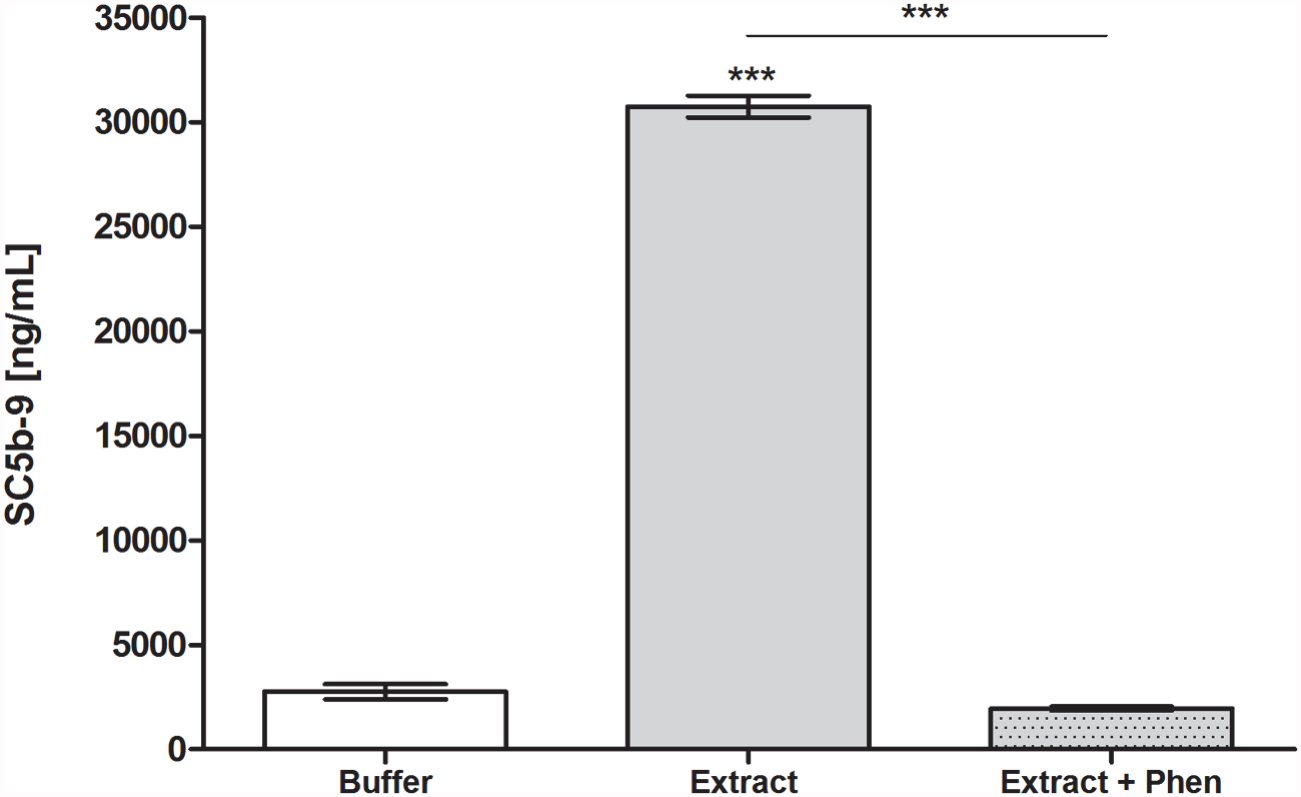
Terminal Complement Complex (TCC) generation induced by the *Premolis semirufa*’*s* bristles extract. Samples of NHS (50 μL) were incubated with the bristles extract (175 μg/mL) or PBS, in the presence or absence of 10 mM 1,10-Phenanthroline (Phen), at 37°C for 30 min, and SC5b-9 complex present in human serum samples was measured using the MicroVue SC5b-9 Plus EIA Kit. Data are representative for two separate experiments, performed in duplicate, and the results are expressed as concentration of SC5b-9 complex per mL of human serum (ng/mL) ± SD. (***) *p* < 0.001: significant differences between the mean values obtained with the buffer and between the treatments.

To evaluate whether activation/consumption of the complement triggered the activation of the Terminal complement pathway, we measured the concentration of SC5b-9 complex present in normal human serum samples incubated with the *Premolis* semirufa’s bristles extract in the presence or absence of 1,10-phenanthroline. As shown in Fig. 3, the bristles extract induced a significant generation of Terminal complement complexes in normal human serum samples that, in turn, was completely inhibited by 1,10-phenanthroline.

### *Premolis semirufa*’s bristles extract directly cleaves purified C3, C4 and C5

The ability of the extract to exert direct proteolytic action on components C3, C4 and C5 was determined by incubating samples of the bristles extract with purified C-components, in the presence or absence of protease inhibitors. The reactions were analyzed by SDS-PAGE for the presence of cleavage fragments.

**Fig 4:**
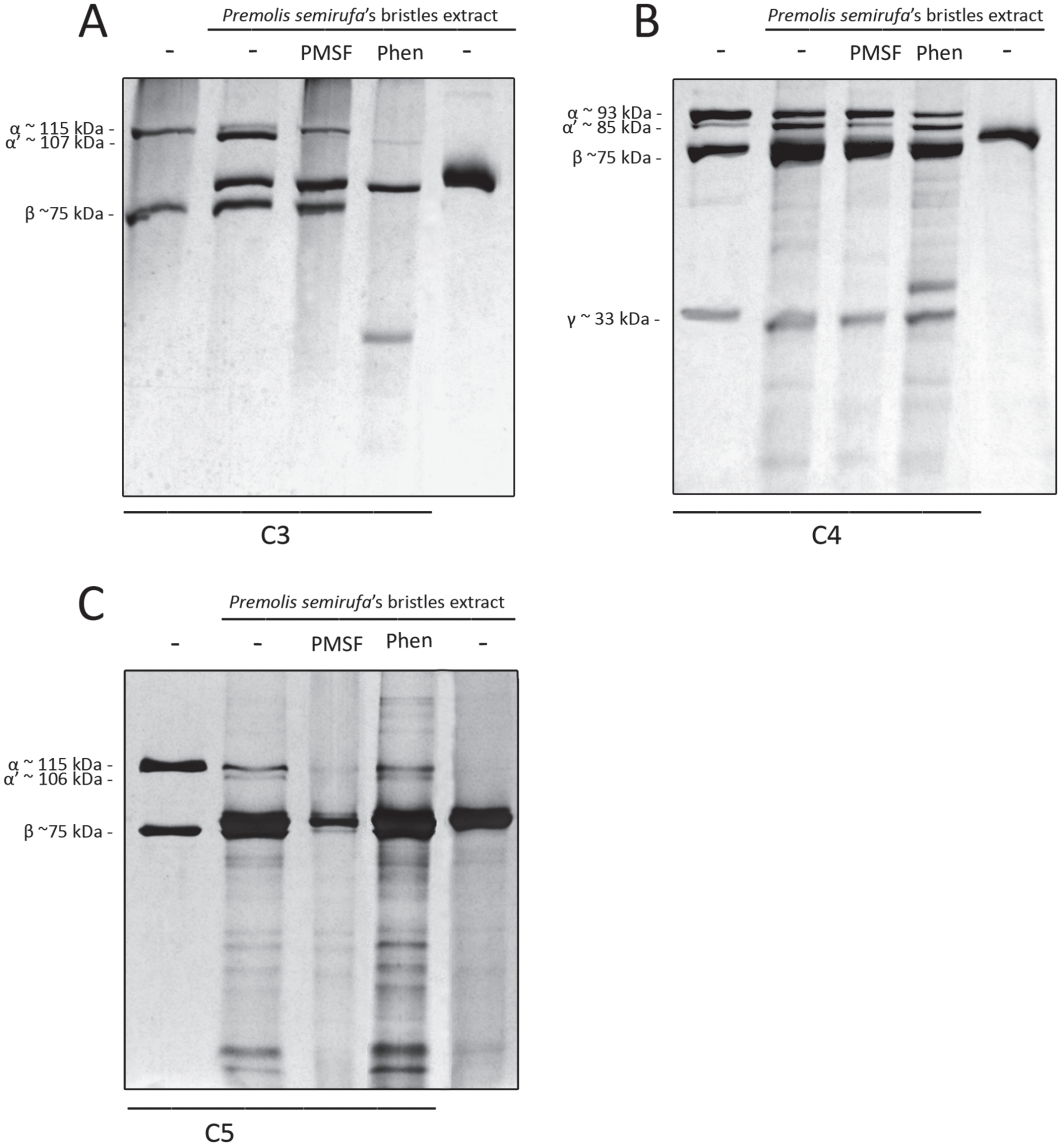
Proteolytic action of the *Premolis semirufa’s* bristles extract on purified human C-components C3, C4 and C5. Samples of the bristles extract (2.0 μg for C3 and C4 or 3.0 μg for C5) were incubated, in the absence or presence of 10 mM PMSF or 1,10-Phenanthroline (Phen), with human C3 (3 μg) **[A]**, human C4 (3 μg) **[B]** and human C5 (3 μg) **[C]** at 37°C for 1 h. Proteolytic activity was examined on 10% polyacrylamide gel under reducing conditions and stained by silver. In the 1^st^ lanes of gels: electrophoretic separation of purified components incubated with PBS as a positive control; 2^nd^ lanes: incubation of purified components with the extract; 3^rd^ lanes: incubation of the mixture with PMSF; 4^th^ lanes: incubation of the mixture with Phenanthroline and 5^th^ lanes: electrophoretic separation of the extract. α (115 kDa) and β (75 kDa) for C3; α (93 kDa), β (75 kDa) and γ (33 kDa) for C4 and α (115 kDa) and β (75 kDa) for C5.

Fig. 4 shows that the extract induced cleavage of the alpha chains of all three evaluated components as indicated by slight reductions (8–9 kDa) in their Mrs, most potently in C3, where nearly all alpha chain was reduced in Mr, while the alpha chains of C4 and C5 seemed to be more resistant (Fig. 4, 2^nd^ lanes). The serine protease inhibitor PMSF inhibited the cleavage of the alpha chains of both C3 and C4 (Fig. 4A and B, 3^rd^ lanes), while the cleavage of the C5-alpha chain seemed to be enhanced. Surprisingly, 1,10-phenanthroline increased the cleavage of the C3 alpha and beta chains caused by the bristles extract (Fig. 4A), but had no effect on the cleavage of C4 and C5 (Fig. 4B and C, 4^th^ lanes). These results suggest a complex role for metalloproteases and serine proteases in the activity of the bristles extract. Of note, the serine protease inhibitor PMSF could not be used in the whole serum assays (Figs. 1–3) as it would also inhibit the serine proteases of the complement system and, thus, would have prevented any complement activation.

### Gel filtration chromatography of the *Premolis semirufa*’s bristles extract

**Fig 5:**
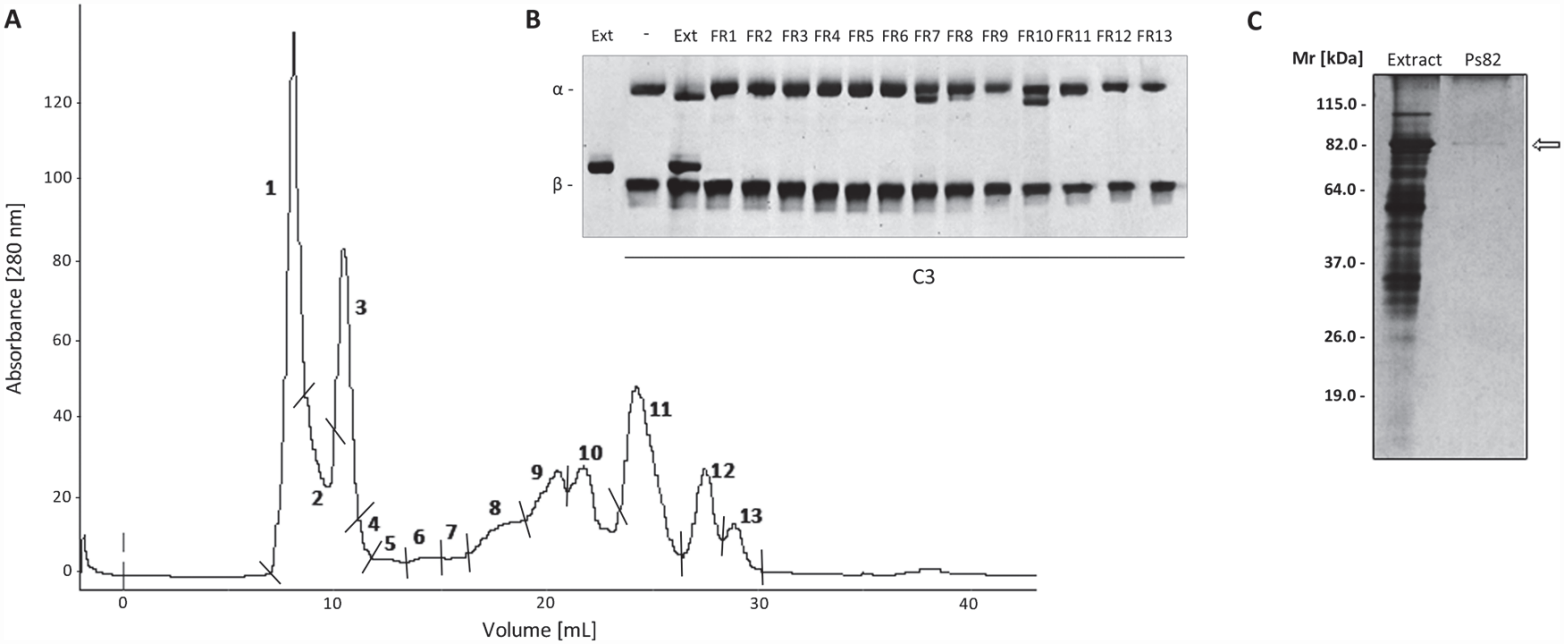
Chromatography of the *Premolis semirufa’s* bristles extract and isolation of the protease responsible for complement activation. **[A]** Chromatography of 1 mg of the extract on a FPLC-GP-250 Plus system using a molecular exclusion column (Superdex 200 10/300 GL), in 0.05 M ammonium bicarbonate buffer, pH 7.4. **[B]** SDS-PAGE of fractions screened by their ability to cleave the component C3. **[C]** SDS-PAGE (12%) of 10 μg of the extract and 0.25 μg of the fraction 10, under reducing conditions, followed by silver staining.

In order to isolate the components acting on the complement system, samples of extract were fractionated using a Superdex 200 10/300GL gel filtration column, which resulted in the elution of 13 chromatographic peaks (Fig. 5A). All fractions were tested for the ability to cleave purified human C3. Fractions 7 and 10 were able to promote this cleavage, as shown in the Fig. 5B, which just the latter fraction showed a single protein band of 82 kDa by SDS PAGE (Fig. 5C). The fraction 10, named here Ps82, was further analyzed as below.

### Analysis of enzymatic properties of Ps82

**Fig 6:**
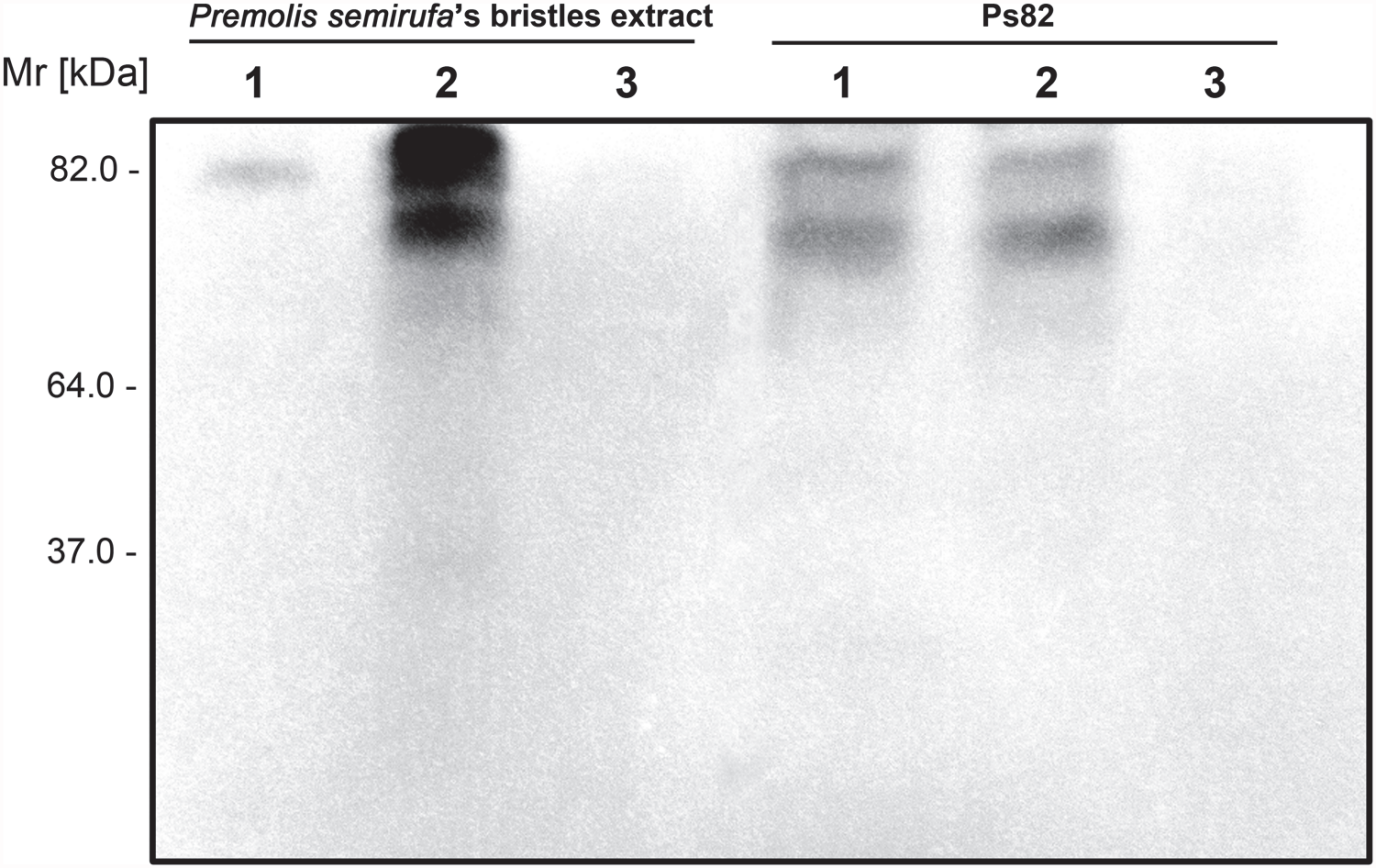
Proteolytic activity of Ps82 analyzed by zymography. Samples of 0.05 μg of Ps82 or 0.5 μg of the extract were incubated in the absence (Lane 1) or presence of 10 mM 1,10-Phenanthroline (Lane 2) or PMSF (Lane 3), inhibitors of metallo- and serineproteases, respectively, submitted to electrophoresis and subsequently incubated, at 37°C for 12 h, in 50 mM Tris-HCl, pH 8.3. Following incubation, the gel was stained with 0.2% Coomassie Brilliant Blue R-250 and the gelatinolytic activity was detected as unstained bands on a dark background. The figure represents the negative of the original image.

We previously showed that the *Premolis semirufa*’s bristles extract, showed strong serine protease activity, both in gelatinolytic zymography, as well as in fluorescence resonance energy transfer (FRET) assays [5], and in the present study we tested the protease activity of Ps82 by zymography (Fig. 6). Samples of 0.05 μg of Ps82 and 0.5 μg of the extract were incubated in the presence or absence of metallo- and serine proteases inhibitors. Crude bristles extract and purified Ps82 both showed gelatinolyitic activity (Fig. 6, lane 1). Gelatinolytic activity of Ps82 and of the bristles extract was completely inhibited by the PMSF (Lanes 3). While addition of the metalloproteinase inhibitor 1,10-phenanthroline had no effect on the gelatinolytic activity of Ps82 (Lane 2), surprisingly it enhanced the gelatinolytic activity of the crude extract (Lane 2).

### Ps82 interferes with the alternative, lectin and classical complement pathways

**Fig 7:**
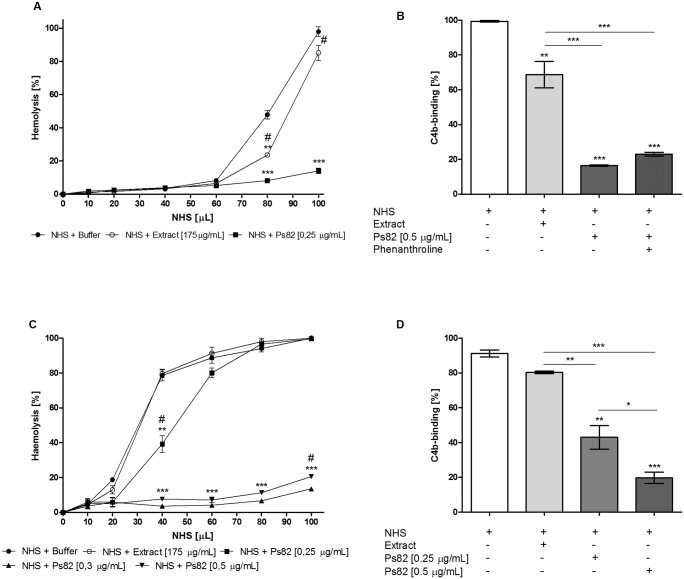
Action of Ps82 on the complement pathways. Samples (50 μL) of normal human serum (NHS) were pre-incubated with 0.25, 0.33 or 0.5 μg/mL of Ps82 or with 175 μg/mL of the *Premolis semirufa*’s bristles extract. The mixtures were pre-incubated for 30 min for the alternative pathway **[A]** or 1 h for the classical pathway **[C]** at 37°C, and then assayed for the residual complement activity in haemolytic tests. For lectin pathway **[B]**, samples of 50 μL of NHS were pre-incubated, in the presence or absence of 10 mM 1,10-Phenanthroline, with 0.5 μg/mL of Ps82 or 175 μg/mL of the *Premolis semirufa*’s bristles extract for 30 min at 37°C and then evaluated for the residual complement activity by ELISA. Alternatively, samples of NHS incubated with Ps82 or the *Premolis semirufa*’s bristles extract were evaluated in conditions for activation of the classical pathway, by the deposition of complement component C4b by ELISA **[D]**. Data are representative for three separate experiments, performed in duplicate, and the results are expressed as percentage of haemolysis (%) ± SD (for A and C) and percentage of C4b deposition (%) ± SD (for B and D) in relation to the control samples (NHS + buffer) and between the treatments. (*) *p* < 0.05 (**) *p* < 0.01 and (***) *p* < 0.001. The symbol (#) indicates significant differences between the extract and Ps82.

Since the *Premolis semirufa*’s bristles extract showed interference with the complement system, the effect of Ps82 on the three C-activation pathways was evaluated. Human serum was pre-incubated with the Ps82 and the remaining complement activity was measured in haemolytic tests and by ELISA. The results show that, similar to the crude extract, although with greater activity at lower concentration, Ps82 also reduced the haemolytic activity of the AP (Fig. 7A) and the LP (Fig. 7B). In addition, but in contrast to the crude extract, Ps82 also interfered with the classical pathway, as shown by the reduction of haemolytic activity and deposition of C4b (Fig. 7C and 7D), thus demonstrating that the pre-incubation with the Ps82 resulted in activation and consumption of the three complement pathways. As expected 1,10-phenanthroline did not significantly interfere with the activity of Ps82 (Fig. 7B). PMSF could not be used in the complement activation assays as it would interfere with the complement serine proteases C1r, C1s, MASPs, C2, fD and fB.

### Ps82 induces generation of C3a and C4a anaphylatoxins, but not C5a or Terminal Complement Complex in human serum

**Fig 8:**
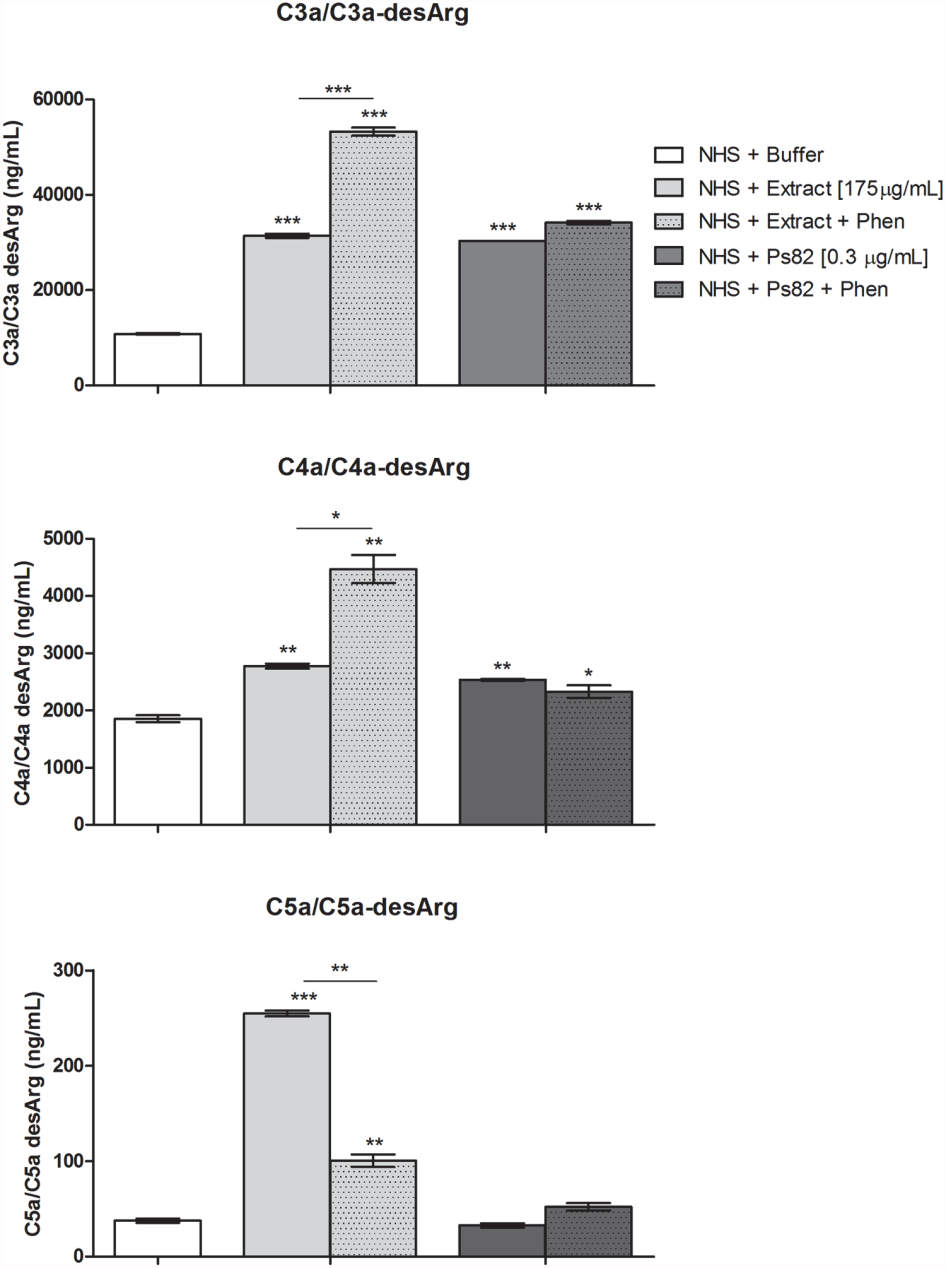
Human serum anaphylatoxins generation induced by Ps82. Samples of NHS (50 μL) were incubated with Ps82 (0.33 μg/mL) or the *Premolis semirufa’s* bristles extract (175 μg/mL), in the presence or absence of 10 mM 1,10-Phenanthroline (Phen), for 30 min at 37°C. The generation of the anaphylatoxins (C3a, C4a and C5a) was measured using the kits "Human C3a ELISA Kit, Human C4a ELISA Kit and Human C5a ELISA Kit". Data are representative for two separate experiments, performed in duplicate, and the results are expressed as concentration of each anaphylatoxin per mL of human serum (ng/mL) ± SD. (*) *p* < 0.05 (**) *p* < 0.01 and (***) *p* < 0.001: significant differences between the mean values obtained with the Buffer and the treatments.

Fig. 8 shows that, similarly to the extract, the Ps82 induced an increase of C3a and C4a generation in human serum. However, in contrast to the *Premolis semirufa*’s bristles extract, the Ps82 did not increase the generation of C5a. Furthermore, while 1,10-Phenanthroline increased the crude bristles extract-induced generation of C3a and C4a and decreased the generation of C5a, the effect of purified Ps82 on anaphylatoxin generation was not affected. Thus, these results corroborate with the data on activation/consumption of the complement system by components present in the *Premolis semirufa*’s bristles extract, and also with the classification of the Ps82 as a serine protease.

In contrast to the *Premolis semirufa*’s bristles extract, the Ps82 did not induce the generation of Terminal complement complexes in normal human serum samples (data not shown).

### Ps82 directly cleaves C components C3, C4 and C5

**Fig 9:**
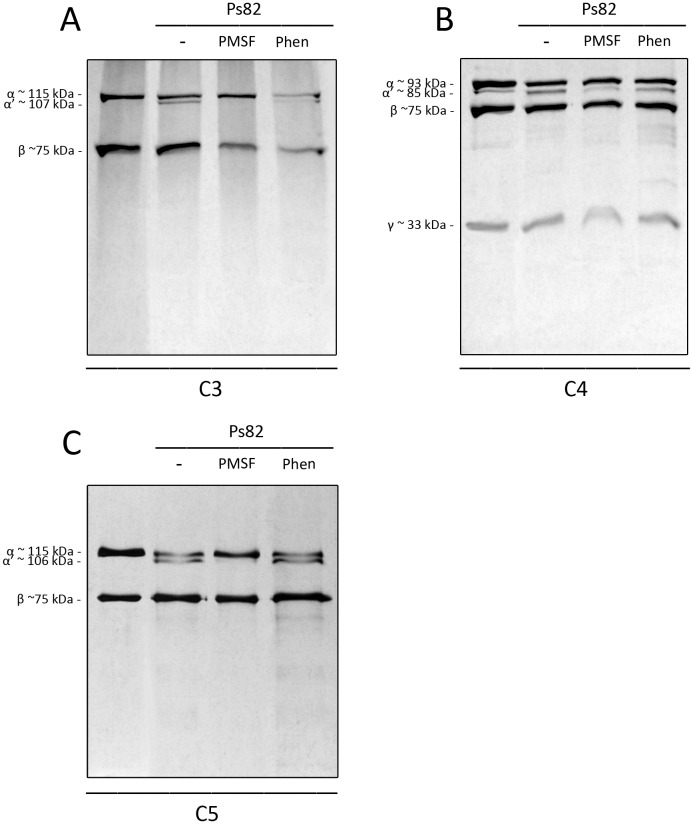
Proteolytic action of Ps82 on purified human C components C3, C4 and C5. Samples of 0.1 μg of Ps82 were incubated, in the absence or presence of 10 mM PMSF or 1,10-Phenanthroline (Phen), with human C3 (3 μg) **[A]**, human C4 (3 μg) **[B]** and human C5 (3 μg) **[C]** at 37°C for 1 h. Proteolytic activity was examined on 10% polyacrylamide gel under reducing conditions and stained by silver. In the 1^st^ lanes of gels: electrophoretic separation of purified components incubated with PBS as a positive control; 2^nd^ lanes: incubation of purified components with Ps82; 3^rd^ lanes: incubation of the mixture with PMSF and 4^th^ lanes: incubation of the mixture with Phenanthroline. α (115 kDa) and β (75 kDa) for C3; α (93 kDa), β (75 kDa) and γ (33 kDa) for C4 and α (115 kDa) and β (75 kDa) for C5.

**Fig 10:**
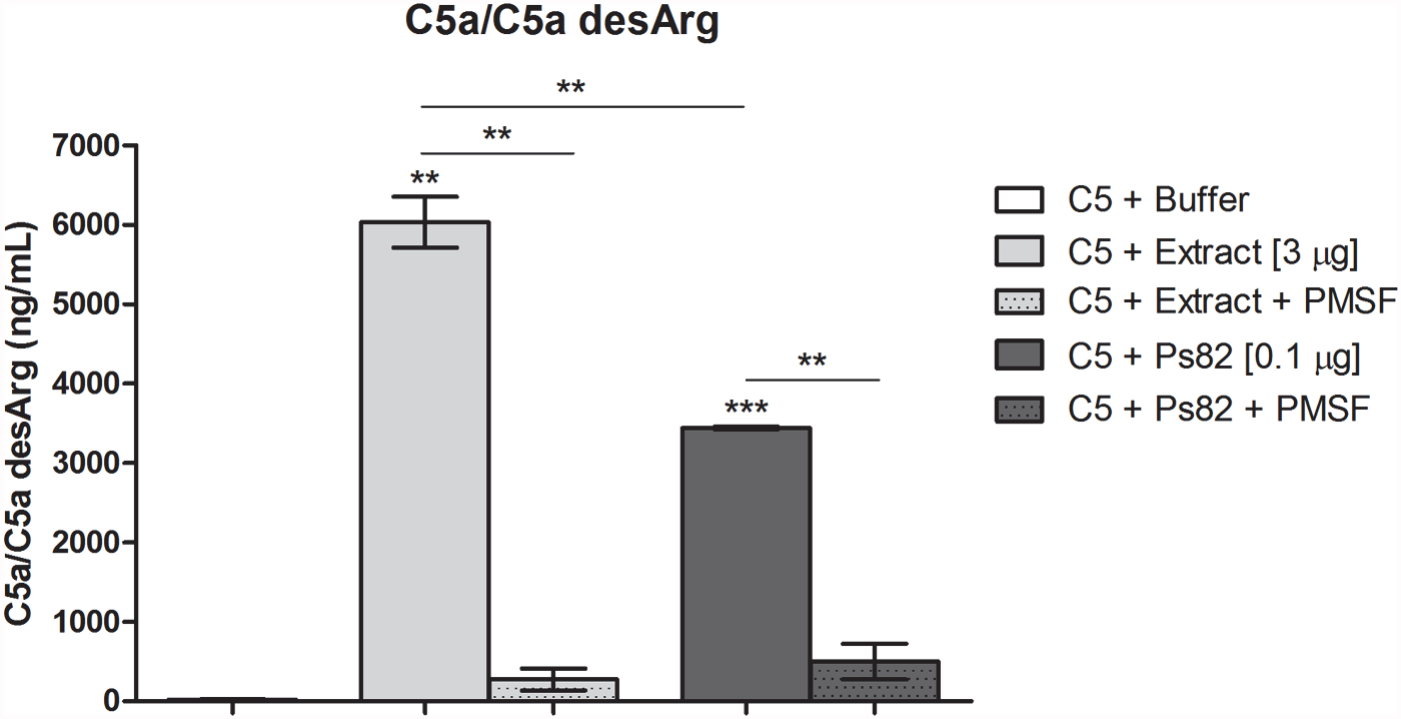
Anaphylatoxins generation from purified human C5 induced by the *Premolis semirufa’s* bristles extract and Ps82. Samples of 3 μg of the extract or 0.1 μg of Ps82 were incubated, in the absence or presence of 10 mM PMSF, with human C5 (3 μg) for 30 min at 37°C. The generation of the anaphylatoxin C5a was measured using the "Human C5a ELISA Kit". Data are representative for two separate experiments, performed in duplicate, and the results are expressed as concentration of anaphylatoxin per mL (ng/mL) ± SD. (**) *p* < 0.01 and (***) *p* < 0.001: significant differences between the mean values obtained with the Buffer and the treatments.

Since the *Premolis semirufa*’s bristles extract promoted direct cleavage of complement components C3, C4 and C5 (Fig. 3), and that the Ps82 was purified based on its direct cleavage of C3 (Fig. 5B), the possible direct cleavage of other components such as C4 and C5 by Ps82 was also analyzed by SDS-PAGE for the presence of cleavage fragments. Fig. 9 shows that incubation of C3, C4 and C5 with Ps82 induced cleavage of the alpha chains of all three components. Moreover, this cleavage was completely inhibited by PMSF, but was not affected by the addition of 1,10-phenanthroline. The beta and gamma (for C4) chains were not affected by Ps82. Concomitantly with the cleavage of the C5 alpha chain, C5a fragments were generated, as detected by ELISA (Fig. 10). This was also inhibited by PMSF.

## Discussion

Studies involving components of animal venoms that interfere with the complement system, acting both by direct cleavage of a specific component or by interacting with elements to form complexes and thereby activate the complement cascade, have been well demonstrated in a variety of animal venoms [19], such as snakes from the families Elapidae, Viperidae and Crotalidae [15, 20–23], spiders [24–26] and scorpions [27]. As an example, cobra venom factor (CVF), the most extensively studied glycoprotein of cobra species of the genus *Naja*, which similarly to the C3b, forms a bimolecular complex CVF/Bb, a C3/C5 convertase that cleaves both complement components C3 and C5 [28]. In addition, there are important human pathogens, such as *Streptococcus pyogenes* and *Staphylococcus aureus*, which express various virulence factors that are able to degrade and inactivate complement components in order to evade eradication by the innate immune system [29, 30].

The presence of components in the venom of caterpillars that interfere with the complement system has not been demonstrated previously. As the complement system has potent pro-inflammatory activities and chronic envenomation by the caterpillar *Premolis semirufa* causes inflammation and the occupational disease ‘Pararama associated phalangeal periarthritis’, it is important to identify the composition of the *Premolis semirufa*’s bristles extract and its possible interference in the human complement system to better understand the mechanisms involved in the disease development.

We show here that incubating normal human serum with *Premolis semirufa*’s bristles extract resulted in consumption of the AP and LP (Figs. 1 and 7) but not of the CP. The observation of complement activation rather than inhibition by the extract has been confirmed by the production of large amounts of the three anaphylatoxins C3a, C4a and C5a (Fig. 2). The presence of the metalloprotease inhibitor 1,10-Phenanthroline in the reaction enhanced the bristles extract induced increase in the generation of C3a and C4a but it inhibited the increase in C5a. As the enzymes of the complement activation pathways are all serine proteases and are not inhibited by 1,10-Phenanthroline, the observation that this metalloprotease inhibitor affected the action of the bristles extract suggested that it may contain enzymes that directly act on the complement system. Thus, the extract may contain a metalloproteinase that either directly cleaves C5, and generating C5a, or the bristles extract activates a metalloprotease in the serum, which then subsequently cleaves C5. In contrast, C3 and C4 seems to be cleaved by protein(s) whose activity is under the partial regulation of metalloproteinase(s), since 1,10-Phenanthroline potentiated C3a and C4a generation in human serum treated with the extract. C5 cleavage could not be completely inhibited by the metalloprotease inhibitor, suggesting that other enzymes are also involved. Anaphylatoxins are potent inflammatory mediators, which can mediate chemotaxis, inflammation, and generation of cytotoxic oxygen radicals [31–33], as well to regulate regeneration and tissue fibrosis [34–36]. Thus, anaphylatoxins play important roles in induction and regulation of inflammation, and the observation, that the caterpillar bristles extract induces all anaphylatoxins, suggest that they may be involved in the chronic and granulomatous inflammation, induction of fibrosis that may progress to joint immobility, as observed in pararamose [3].

That the *Premolis semirufa*’s bristles extract caused activation rather that inhibition of the complement system was also confirmed by the observation of the Terminal complement complex (TCC/SC5b-9) generation in NHS incubated with the extract. SC5b-9 formation was completely inhibited by the presence of 1–10 Phenanthroline. Analysis of a possible direct proteolytic action of the *Premolis semirufa*’s bristles extract on the complement molecules showed that the extract induced cleavage of the alpha chains of C3, C4 and C5 (Fig. 4), possibly contributing to the activation of the complement pathways, as well as to the generation of the anaphylatoxins. The addition of the metalloprotease inhibitor 1,10 Phenanthroline did not inhibit the cleavage of any of the components, however the serine protease inhibitor PMSF clearly inhibited the C3 and C4 proteolysis, demonstrating that the extract also contains serine protease activity. However, PMSF enhanced the cleavage of C5, suggesting a very complex interactions and regulations of proteases in the extract.

In order to identify the protease that is responsible for the cleavage of the complement components, we fractionated the bristles extract by gel filtration and tested the fractions on their ability to cleave C3. We identified two fractions with C3-cleaving ability, the most potent of which contained a protein with Mr around 82 kDa, named here Ps82 (Fig. 5). We further characterised this fraction and showed that activity was caused by a serine protease, which also had gelatinolytic activity (Fig. 6), activated all three C-activation pathways (Fig. 7), generated anaphylatoxins (Figs. 8 and 10) and cleaved purified C3, C4 and C5 components (Fig. 9). Cleavage of C3, C4 and C5 and gelatin were all inhibited by PMSF suggesting that Ps82 is a serine protease. In contrast to the crude bristles extract, Ps82 also inhibited the lytic activity of the classical pathway, but unlike the extract, it was not able to generate C5a and Terminal complement complex (TCC), in human serum.

In contrast to the inability of Ps82 to generate C5a in whole serum, Ps82 generated the anaphylatoxin C5a by direct cleavage of purified C5, suggesting that Ps82 can have higher affinity/specificity to other molecules existing in the human serum, which may result in a reduced availability of this enzyme for C5 cleavage. Further studies investigating the affinity of Ps82 on the complement components will be conducted.

Considering that some of the activities of the crude extract could be either inhibited or enhanced by the metalloprotease inhibitor 1.10-Phenanthroline, suggest that the extract also contains metalloproteases. The isolation and characterization of these is subject to further studies.

Gelatin zymography assays demonstrated that the presence of Phenanthroline enhanced the gelatinolytic activity of the extract and promoted the appearance of other bands, suggesting the existence of metalloprotease(s) with regulatory role on the serine protease(s) activity, since the use PMSF completely blocked the activity. The purified Ps82, that on the silver stained SDS-PAGE gel showed a single band around 82 kDa (Fig. 5), presented in the zymography assay with two bands with strong gelatinolytic activity, one of approximately 82 kDa and other of slightly smaller mass, both of which were completely inhibited by PMSF. This result may indicate the presence of isoform in the purified fraction or some fragmented protein.

In conclusion, our data demonstrate that the *Premolis semirufa’s* bristles extract can activate the complement system, generating biologically active fragments, such as anaphylatoxins, which can play an important role in the inflammatory process presented in humans after envenomation. The use of 1,10-Phenanthroline and PMSF in the assays showed that the extract contains a complex mixture of proteins with metalloprotease and serine protease activities which can interact with the complement system and some of which regulates the other’s activities. We identified a serine protease containing fraction (named here Ps82), which we showed has potent complement activating activities, similar to that of the whole extract. Further identification and characterization of this compound may lead to future therapies for the prevention and treatment of Pararamose.
